# Comprehensive Analysis of Hormone Formulations and Emotional Effects in Transgender Men Undergoing Hormone Replacement Therapy

**DOI:** 10.1192/j.eurpsy.2025.1545

**Published:** 2025-08-26

**Authors:** A. Stewart, B. Carr, L. Schmidt

**Affiliations:** 1 College of Allopathic Medicine, Nova Southeastern Univeristy, Ft. Lauderdale; 2 Psychiatry, University of Florida, Gainesville, United States

## Abstract

**Introduction:**

Hormone replacement therapy (HRT) for transgender men involves various formulations of testosterone, each exhibiting unique pharmacokinetic profiles and emotional impacts. A comprehensive understanding of these variations is crucial for optimizing treatment outcomes and managing side effects effectively.

**Objectives:**

This study aims to elucidate the differential emotional effects associated with various testosterone formulations used in HRT for transgender men.

**Methods:**

A comprehensive literature review was conducted using databases such as PubMed, Scopus, and Web of Science. The review focused on four primary testosterone administration methods: intramuscular and subcutaneous injections, transdermal patches and gels, oral testosterone, and implantable pellets. Key metrics evaluated included pharmacokinetics, emotional effects, and nature of side effects.

**Results:**

The review identified distinct pharmacokinetic profiles and emotional responses associated with each testosterone formulation:

Intramuscular and Subcutaneous Injections: These methods showed peak testosterone levels within 24-48 hours, followed by a decline over one to two weeks. Emotional effects included mood elevation and increased energy during the peak phase, with potential irritability or anxiety. The trough phase was marked by mood swings and depressive symptoms, particularly before the next injection. Side effects such as acne and libido changes peaked shortly after injection and decreased before the subsequent dose.

Transdermal Patches and Gels: These methods maintained consistent blood testosterone levels, resulting in stable mood and emotional states with reduced mood swings. Initial therapy adaptation caused mild mood changes, and side effects were primarily localized to skin irritation at application sites.

Oral Testosterone: Testosterone undecanoate offered stable testosterone levels with consistent mood regulation. Gastrointestinal side effects were common, and emotional stability varied based on absorption rates and adherence to dosing schedules.

Implantable Pellets: These provided the most stable testosterone levels over several months, leading to very stable emotional states with minimal mood fluctuations. Side effects included localized reactions such as discomfort or infection at the implantation site, with minimal systemic side effects.

**Conclusions:**

The choice of testosterone formulation significantly impacts the emotional well-being of transgender men undergoing HRT. Intramuscular and subcutaneous injections were associated with emotional fluctuations tied to hormone peaks and troughs, while transdermal, oral, and implantable methods provided more stable hormone levels and emotional states. Regular monitoring and individualized modifications are crucial to optimizing physiological and emotional outcomes and enhancing the quality of life for transgender men.

**Disclosure of Interest:**

None Declared

